# Supplementary material for: Hybrid Models and Biological Model Reduction with PyDSTool
Source: PLoS Comput Biol. 2012 Aug 9;8(8):e1002628. doi: 10.1371/journal.pcbi.1002628 (PMC3415397; doi:10.1371/journal.pcbi.1002628)
Supplement: Text S4 — Complete source code for the PyDSTool package (version 0.88.120504). Includes API documentation and help files linking to web pages. This file is identical to the current public release on Sourceforge.net. (ZIP) [file pcbi.1002628.s004.zip › PyDSTool/html/toc-PyDSTool.common-module.html]

xml version="1.0" encoding="ascii"?


common


# Module common

---

## Classes

API\_class

BarycentricInterpolator  
 DefaultDict  
 Diagnostics  

DomainType

KroghInterpolator  
 PiecewisePolynomial  
 Struct  
 Utility  
 Verbose  
 and\_op  
 args  
 fit\_cubic  
 fit\_diff\_of\_exp  
 fit\_exponential  

fit\_function

fit\_linear  
 fit\_quadratic  
 fit\_quadratic\_at\_vertex  
 interp0d  
 interp1d  
 interpclass  
 metric  
 metric\_L2  
 metric\_L2\_1D  
 metric\_float  
 metric\_float\_1D  
 metric\_weighted\_L2  
 metric\_weighted\_deadzone\_L2  
 not\_op  

null\_predicate\_class

or\_op  
 predicate  

predicate\_op

## Functions

\_scalar\_diff

array\_bounds\_check  
 arraymax  
 className  
 compareBaseClass  
 compareClassAndBases  
 compareNumTypes  
 concatStrDict  
 copyVarDict  
 diff  
 diff2  
 ensurefloat  
 extent  
 filteredDict  
 getSuperClasses  
 get\_opt  
 idfn  
 insertInOrder  

intersect

invertMap  
 isUniqueSeq  
 isincreasing  
 ismonotonic  
 linearInterp  
 listid  
 makeArrayIxMap  
 makeMultilinearRegrFn  
 makeSeqUnique  
 makeUniqueFn  
 make\_poly\_interpolated\_curve  
 n\_sigdigs\_str  
 nearest\_2n\_indices  
 noneFn  
 object2str  

remain

simple\_bisection  
 simplifyMatrixRepr  
 smooth\_pts  
 sortedDictItems  
 sortedDictKeys  
 sortedDictLists  
 sortedDictValues  
 timestamp  
 uniquePoints  
 verify\_intbool  
 verify\_nonneg  
 verify\_pos  
 verify\_values  

## Variables

API  
 Continuous  
 Discrete  

Inf

LargestInt32  

Macheps

NaN

\_all\_complex  
 \_all\_float  
 \_all\_int  
 \_all\_numpy\_complex  
 \_all\_numpy\_float  
 \_all\_numpy\_int  

\_classes

\_complex\_types  

\_constants

\_float\_types  

\_functions

\_int\_types  

\_mappings

\_num\_equivtype  
 \_num\_maxmin  
 \_num\_name2equivtypes  
 \_num\_name2type  
 \_num\_type2name  
 \_num\_types  
 \_pytypefromtype  
 \_real\_types  
 \_seq\_types  

\_typefrompytype

\_verify\_type\_names

complex192  

exp

float96  

greater

isfinite

less

less\_equal

log

logical\_or

null\_predicate  

sign

targetLangs  

---

[hide private]
